# Perspectives on Sleep, Sleep Problems, and Their Treatment, in People with Serious Mental Illnesses: A Systematic Review

**DOI:** 10.1371/journal.pone.0163486

**Published:** 2016-09-22

**Authors:** Sophie Faulkner, Penny Bee

**Affiliations:** 1 The School of Nursing, Midwifery and Social Work, University of Manchester, Manchester, United Kingdom; 2 Manchester Mental Health and Social Care Trust, Manchester, United Kingdom; University of Regensburg, GERMANY

## Abstract

Sleep problems are common in people with serious mental illness, and impact negatively on functioning and wellbeing. To understand the development of sleep problems, their maintenance, and their treatment, an in depth understanding of patient perspectives is crucial. A systematic literature review was conducted using Medline, AMED, PsychInfo, Embase and CINAHL. Qualitative and quantitative studies were included if they explored or measured patient perspectives on sleep, sleep problems or sleep treatments in people with serious mental illness. Of the 2,067 hits, only 22 met review inclusion criteria, and high quality evidence was sparse. The limited findings suggested sleep was seen as highly interlinked with mental health. Evaluations of treatments varied, however perceived efficacy and personalisation of treatments were valued. Some evidence suggested patient priorities and conceptualisations regarding sleep may diverge from those of validated screening tools developed in general population and sleep medicine samples. More rigorous research is needed to support adaptation and development of interventions and outcome measures for use in specialist mental health settings. Qualitative studies exploring the experience of sleep disturbance in particular diagnostic groups and contexts are urgently required, as are patient perspectives on sleep interventions.

## Introduction

Sleep disturbances are the most commonly reported psychiatric symptom in the general population [1], with rates of insomnia ranging from 6% to 30% depending upon the definition adopted [2]. The prevalence of sleep disturbances is substantially higher in people with mental illnesses [3]. Sleep disturbances are a core biological symptom of depression [4], a relapse indicator in bipolar [5], and are present in up to 80% of those with psychotic illnesses [6], often persisting after other symptoms have been treated [7].

Researchers are just beginning to understand the complex interconnections between the neurobiological mechanisms underlying various psychiatric diagnoses, and the mechanisms of sleep [3,8]. These interactions offer to explain this frequent co-occurrence of sleep problems with mental illnesses. Importantly, it is well established that co-morbid sleep problems impact negatively on the course of psychiatric illness, and on recovery and quality of life [9,10].

Serious Mental Illness (SMI) is often considered to include diagnoses which most commonly require treatment from specialised services (secondary care), such as psychotic illnesses and bipolar affective disorder. Despite its known impact, sleep disturbance remains poorly understood and neglected in people with SMI [8], and is thought to be underdiagnosed in this population [11]. This may be partly attributable to long-standing conceptualisations distinguishing ‘primary’ sleep disturbances, from sleep disturbances ‘secondary’ to mental health conditions [12]. The utility and reality of this distinction has been questioned [13], as has the assumption that sleep disturbances will automatically resolve with treatment of other symptoms. Epidemiological evidence suggests sleep disturbances in fact more often pre-date, than are a by-product of, other symptoms [14]. Contemporary illness classifications have removed this primary/secondary distinction, emphasising the need for independent clinical attention to sleep disturbance irrespective of co-morbidity [15]. It is therefore important that relevant research is identified to support this clinical area.

Insomnia without significant co-morbidity has been relatively well studied to date, both through objective means including polysomnography [16], and through qualitative exploration of patient’s perspectives and experiences [17–19]. A range of treatments for insomnia have been evaluated; clinical trials support the efficacy of Cognitive Behavioural Therapy for Insomnia (CBT-i) and related approaches [20]. Thus evidence based treatment recommendations [21,22] and best practice guidance [23] now exist. In many cases, however, these studies excluded people with SMI either purposefully through study exclusion criteria, or as a result of recruitment contexts [24]. Generalisability cannot be presumed. Treatments are increasingly beginning to be adapted and tested in some SMI populations with some early signals of success [10,25], but these are not yet routinely implemented and their acceptability is not well established.

Patient perspectives make an important contribution toward developing better understanding of the relationships between sleep and SMI, particularly qualitative accounts which are recommended to inform early theoretical development [26]. Health behaviour theories, although diverse, agree that beliefs and attitudes influence human behaviour [27]. Patient perspectives are therefore particularly important in the design of behavioural and psychological interventions, which rely so much on the patient’s motivation to adhere to prescribed behavioural modifications and to be actively engaged in therapy. The consideration of user perspectives is recommended by the Medical Research Council as a crucial stage in the design and optimisation of complex interventions [28].

### Purpose of the current review

This review systematically examines experiences and perspectives of people with SMI regarding sleep disturbance and its treatment, establishing the size and credibility of the current evidence base, and current evidence gaps.

## Methods

### Search strategy

Systematic searches were performed using both keywords and subject headings in five major databases, search terms included truncation (*) and wildcards where relevant to accommodate different word variants (S1 Table). The broad search concepts were:

- sleep or sleep problems (e.g. ‘circadian’, ‘*somnia’)
          AND
- mental illness and specific included diagnoses (e.g. ‘psychiatric’, ‘bi-polar’)
          AND
- service user perspectives (e.g. ‘beliefs’, ‘self-report’, ‘focus groups’)

Searches were limited to human studies and English language, all searches were conducted from database inception. Searches were run between March and July 2015, and updated in December 2015 (resulting in two new inclusions). Results were saved, imported into reference management software, de-duplicated, and screened by the first author. Database searches were supplemented by reference checking of included articles, and related reviews, and by contacting key authors in the field.

### Screening and inclusion criteria

The definition of SMI is controversial and is sometimes defined in relation to the impact of the condition on functioning, as opposed to diagnosis [29]. For the purposes of this review, an inclusive user-centred definition of SMI, was adopted [30]. This included psychotic illnesses, severe affective disorders, and personality disorders, and aimed to include the main diagnostic groups treated within secondary care (specialist) mental health services. See Table 1 for detailed criteria followed in determining inclusion of studies by participants. To ensure rigour in the review process, these criteria were collaboratively defined and borderline cases were discussed between two researchers to reduce selection bias.

**Table 1:** Detailed participant Inclusion Criteria.

**Age:**	- Participants were adults (over 18), or over 50% were adults.
**Proportion of participants with SMI:**	- Over 50% of participants had SMI. - Studies with lower percentages of people with SMI were included only if results for this subgroup were clearly distinguishable from the results for other participants or groups. - If no figures were given but the term ‘most’, or equivalent, was used the study was included.
**Defining SMI:**	- Diagnoses included within ‘SMI’ were: schizophrenia, other psychotic illnesses, borderline personality disorder and other personality disorders, severe affective disorders including severe unipolar depressive disorder, psychotic depression, severe postnatal depression and puerperal psychosis. - To define severe unipolar depression, criteria were also modelled on those used by Bee et al. [30]; cases were included where psychiatric diagnosis, or a screening tool, indicated severe depression. If this information was not available severity was judged by terms used to describe the condition (e.g. ‘treatment resistant’).
**Inclusion by recruitment setting:**	- Where conditions were unspecified (beyond ‘psychiatry in/outpatients’) studies of samples in secondary mental health care were included, whilst primary care studies were excluded.

Articles were included if they provided primary research data relating to an understanding of the nature of subjective attitudes, thoughts and beliefs around sleep disturbance and its treatment in people with SMI. Attitudes and perceptions may be explored qualitatively, such as through interviews and focus groups, resulting in a narrative analysis, or measured quantitatively, such as through self-report measures and attitude rating scales, resulting in numerical data. Both qualitative and quantitative designs were included in the current review.

Studies were included if they explored perspectives, attitudes, or patient priorities, regarding normal sleep, sleep problems, or sleep treatments. This could be as a primary or secondary objective. Studies describing self-reported ratings of sleep quality or sleep behaviour, but which did not describe participants’ perspectives or interpretations were not included. Sleep problems could include difficulties with sleep initiation, maintenance, timing, or unrefreshing sleep. Studies focused solely on sleep disordered breathing (including sleep apnoea), sleep related movement disorders (including restless leg syndrome) and parasomnias (including sleep walking) were excluded.

Only studies published in peer-reviewed journals were included. This included brief reports of empirical data, but excluded dissertations and conference abstracts, these authors were contacted to seek subsequent peer-reviewed publications.

After screening against selection criteria, all included studies were read and summarised in the study overview tables shown in S2 Table. Data on study population, setting, aim, methods, and findings relevant to the review question were extracted into standardised data extraction pro-forma by the lead author. Key findings were qualitatively compared between and within diagnostic groups, and findings were organised into themes.

### Appraisal of methodological quality

Methodological quality was appraised by the first author during synthesis. Qualitative studies were appraised using criteria from Kuper et al. [31] regarding sample, data collection, analysis, and reflexivity. Included quantitative studies used diverse research designs, but their measurement of attitudes and perceptions was the focus within this review. Quality criteria for quantitative studies therefore focused predominantly on the validity and reliability of measurement of attitudes and beliefs. Quantitative appraisal was achieved through use of published guidance for papers reporting questionnaire research [32], supplemented by criteria from Acaster et al. [33], and from Mokkink et al.'s [34] work on Patient Reported Outcome Measures (S1 File).

## Results

**Fig 1:**
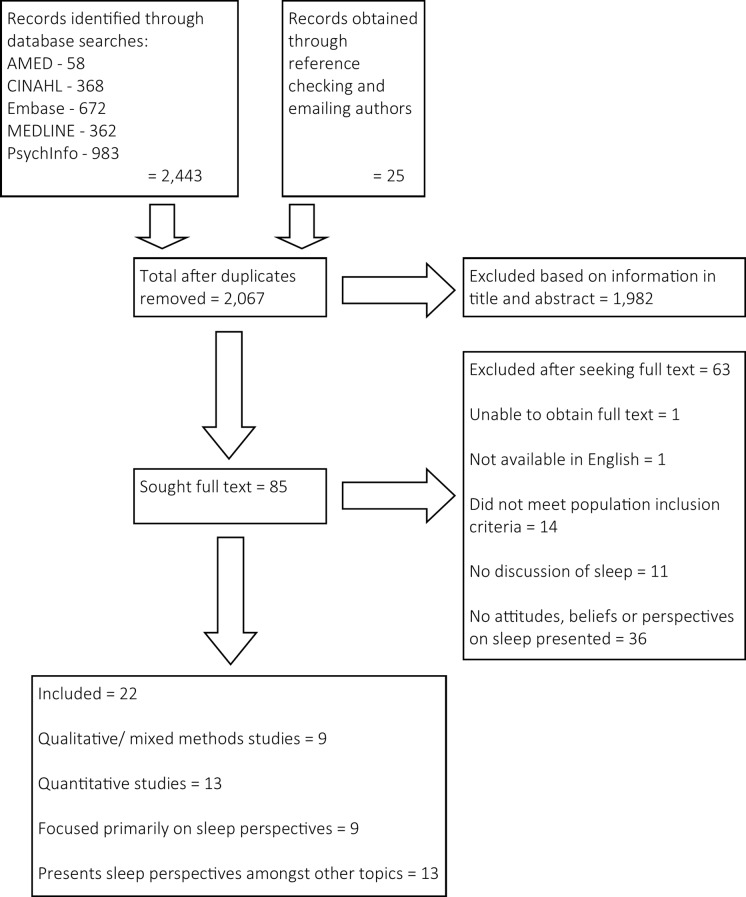
Flow of articles through search and screening process.

A comprehensive search strategy returned 2,066 potentially relevant articles, of which 22 were included in the synthesis. As per PRISMA guidance, Fig 1 shows the flow of articles through the search and screening process [35].

### Overview of results

Of the twenty-two included studies, nine were qualitative and thirteen were quantitative. Quantitative studies included studies which: reported prevalence of participant opinions (n = 2), ranked the comparative importance of concepts (n = 5), compared results of multiple measures within a single group at one time point (n = 3) or multiple time points (n = 1), compared measures between defined groups of participants (n = 2). Table 2 gives an overview of the populations, settings and design of included studies. For more detailed study summaries please see S2 Table. It is worth noting that the majority addressed sleep perspectives only as a secondary objective.

**Table 2:** Summary of populations, methodologies and focus of included studies.

Population		Qualitative Studies	Quantitative studies
**Schizophrenia spectrum disorders**	Inpatients	-	• Chiu et al. 2015* • Lien et al. 2003
	Outpatients	• **Waite et al. 2015**	• Auslander & Jeste 2002 • Poulin et al. 2010
	Mixed settings	• **Waters et al. 2015**	-
**Post-partum psychosis**	Inpatients	-	-
	Outpatients	• Engqvist et al. 2011 • Engqvist & Nilsson 2013	-
	Mixed settings	-	-
**Mixed SMI**	Inpatients	• **Collier et al. 2003**	-
	Outpatients	**•** Davis & O’Neill 2005 • **MacDonald et al. 2015**	**•** Mueser et al. 1992 • **Sobieraj et al. 2013**
	Mixed settings	• **Holmes et al. 1995**	• **Niet de et al. 2008** • **Peacey et al. 2012**
**Bipolar**	Inpatients	-	-
	Outpatients	• Samalin et al. 2014	• Harvey et al. 2005
	Mixed settings	-	-
**Borderline personality disorder**	Inpatients	-	-
	Outpatients	-	• Plante et al. 2013
	Mixed settings	-	-
**Depression**	Inpatients	-	• Li et al. 2011
	Outpatients	-	• Zimmermann et al. 2013
	Mixed settings	-	• Pandina et al. 2010

* **Bold text** = sleep related perspectives were a primary focus, non-bold text = presenting sleep related perspectives was a secondary objective.

Table 2 shows a slight tendency toward quantitative studies despite the topic of patient perspectives being perhaps more naturally addressed using qualitative methods. The largest number of studies focused on this topic used mixed SMI samples, there was also a recent spike in relevant studies in schizophrenia spectrum disorders (3 studies dated 2015). Many complete gaps in evidence are shown, including the absence of any qualitative studies with participants with severe depression or borderline personality disorder.

Understandably, there was a tendency toward studies recruiting patients who were mentally stable (not acutely unwell) at the time of participation, and more studies recruited outpatients than inpatients. Studies in schizophrenia spectrum disorders and in mixed SMI, did however often include participants at various phases of illness, including inpatients.

Five themes emerged from the data: 1) Beliefs about the impact of sleep problems, and the importance of sleep; 2) Sleep expectations and priorities; 3) Perceptions regarding factors which negatively affect sleep; 4) Experiences of others’ attitudes toward sleep problems; 5) Perspectives on approaches to improve sleep.

### Methodological quality of included studies

As per Cochrane guidance [36], summary ratings of the quality of included studies are presented itemised in S3 Table. Articles of poor quality were not excluded, although sources of potential unreliability are discussed during synthesis, and highlighted in these summary tables.

Overall quality of the qualitative studies was mostly medium or good, although some suffered from problems with descriptiveness of analysis or overreliance on reporting the prevalence of opinions in small qualitative studies. In some cases appraisal suggested biasing influences, such as the likely action of a social desirability bias during data collection.

The quantitative studies were highly variable in quality, and in size, although the vast majority were of medium or good quality. Frequently problems were identified with the validity of some measures used, making it unclear what participants might have meant by the answers they had selected, which affected interpretation. The administration of measures was done well in the cases where it was reported on, however it was often not reported on, raising doubts regarding the adequacy of the administration of these measures in these studies. It is important to note that the appraisals reported relate only to the aspects of methodology and content relevant to this review, which for some studies was a smaller part of their overall aim.

### 1) Beliefs about the impact of sleep problems, and the importance of sleep

#### Impacts of poor sleep–qualitative evidence

Those with mixed SMI interviewed in MacDonald et al. [37] cited the impact on functioning as the main reason it was important to treat sleep problems pharmacologically. In a study recruiting those with psychosis [38] participants expressed the same idea, and emphasised the impact of poor sleep on functioning, whilst others felt they carried on as normal. There was limited exploration of the nature of this perceived impact on functioning.

There was an emerging consensus that people with SMI perceive poor sleep to impact significantly on mental health: Participants with psychotic and affective disorders with co-morbid substance misuse felt improved sleep routines helped prevent relapse [39], and some participants stated or implied that lack of sleep caused the onset of their postpartum psychosis [40]. Participants with mixed SMI in MacDonald et al. [37], and with psychotic illnesses in Waite et al. [38], described sleep as significantly impacting on their mental health. Going further, participants with mixed SMI in Collier et al. [41] felt sleep problems were so strongly interlinked with their mental health diagnosis, that they could sometimes be indistinguishable.

#### Impacts of poor sleep—quantitative evidence

One quantitative study surveyed particular perceived impacts of poor sleep, showing some agreement regarding the perceived impact on mood and mental wellbeing described above; forty-nine psychiatry outpatients reported poor sleep impacted on mood, caused tiredness, low energy and motivation. However despite these impairments, few reported any impact on social and work functioning [42]. This was the only quantitative study to report specific perceived consequences of poor sleep.

The degree of perceived negative consequences of poor sleep were also measured by the Dysfunctional Beliefs and Attitudes about Sleep (DBAS) tool, in the ‘Consequences’ subscale, it being thought that exaggerated perception of negative consequences of poor sleep is dysfunctional. The scale’s statements are: “Insomnia interferes with functioning. Mood disturbances due to insomnia. Cannot function without a good night. Lack of energy due to poor sleep. Cancel obligations” [43] (p1551). Higher scores can be driven by increased perception of impact on mood, on functioning, or both. Many studies used the DBAS but only two reported the ‘consequences’ subscale separately to other subscales, and neither itemised for these individual statements. Scores on this scale were higher in less recovered patients with borderline personality disorder, than in more recovered patients [44]. It might be expected that ‘dysfunctional’ exaggerated perceptions of negative consequences of poor sleep might be associated with higher levels of mental illness, however in Li et al. [45] the opposite was found, with perceived negative consequences of poor sleep, rated on the DBAS, increasing rather than decreasing after successful treatment of depression. It is possible this finding may have reflected reduced acceptance of poor sleep as mood improves, rather than a worsening of sleep with improvement in mood, however further research is needed to replicate and to explore the meaning of this finding.

#### Relative importance of sleep

Two studies examining patient priorities in treatment of depression asked about the relative importance of sleep problems compared to other symptoms. In Zimmermann et al. [46] and Pandina et al. [47] large samples with depression ranked which symptoms were most troubling (n = 227 and n = 268 respectively). Both reached the conclusion that sleep symptoms were not the *most* troubling, but were amongst the *more* troubling symptoms one could experience; 52.2% ranked ‘reduced sleep’ the fourth most troubling symptom [47], while ‘sleep disturbance’ influenced ratings of wellbeing moderately, more-so than ‘feelings of guilt’ or ‘depressed mood’ [46]. Terminology should be noted: ‘*reduced* sleep’ may not capture attitudes to altered sleep quality, timing or refreshing-ness, and does not capture dissatisfaction with excessive sleep, whilst ‘sleep *disturbance’* might be interpreted by some to specifically mean *interrupted sleep*.

In a study of a similar aim [48], participants with psychotic disorders endorsed the statement ‘I would like to sleep better’ as the sixth most significant of 50 wellbeing and functioning statements when surveyed (p396). This phrase appears more inclusive of all sleep parameters, and perhaps captures desirability of good sleep, rather than tolerability of poor sleep. No qualitative studies discussed the relative importance of sleep compared to other areas of wellbeing or symptoms, instead focusing on why sleep was perceived as important in relation to the experience of poor sleep or its perceived consequences.

#### Summary

Overall triangulation of the limited evidence available suggests sleep is seen as reasonably important, and that the lack of adequate sleep is recognised to have some negative consequences, although the evidence inadequately describes which consequences are most widely perceived. Impacts on mental wellbeing or on functioning were however cited as reasons to value adequate sleep.

### 2) Sleep expectations and priorities

#### Quantitative evidence

Sleep satisfaction may not depend on just the factors assessed in clinical screening or laboratory measurement. Niet De et al. [49] found, through logistic regression, that only certain Pittsburg Sleep Quality Index (PSQI) questions reflected psychiatric patients’ perception of a sleep problem. Impact on daytime functioning contributed to perception of a sleep problem, as did waking in the night or early morning, or bad dreams. Waking to urinate and sleep latency (time to fall asleep) were not predictive of perceiving a sleep problem [49], suggesting these did not trouble people as much.

Poulin et al. [50] found similarly that sleep latency was significantly higher in patients with schizophrenia compared to healthy controls, yet sleep satisfaction was similar between groups. It is important to note however that sleep satisfaction was only compared between groups, potentially missing within-group relationships. Furthermore, satisfaction was measured through a dichotomous yes/no response [50], which may not have detected subtle differences.

#### Qualitative evidence

Waite et al.’s [38] participants with psychotic illnesses give qualitative descriptions of sleep problems; from language used reasonable inferences can be made regarding negative attitudes to some of these experiences. Frustration, worry, broken sleep, and nightmares were described as troubling, but perhaps reflecting agreement with quantitative findings above [49], taking a long time to fall asleep was not discussed. Collier et al. [41] describe participant’s with mixed SMI’s wish for ‘a good night’s sleep’, however what constituted good sleep was not further explored.

#### Summary

The quantitative evidence, although minimal, highlights potential differences in the priorities and expectations of people with SMI regarding sleep. The fact that no qualitative studies described an aim of exploring sleep expectations, priorities or desires, presents a gap in the literature. Discussions may have relied on assumptions about what is valued. Researchers’ assumptions could be based on research in other populations, or even the temptation to assume one’s own priorities and preferences, about something as ubiquitous as sleep, are universal.

### 3) Perceptions regarding factors which negatively affect sleep

#### Perceived impact of psychiatric and psychological factors–qualitative evidence

Participants with mixed SMI in Collier et al.'s qualitative study [41] and in Holmes et al.'s [51] mixed methods study, described the impact of mental health conditions on sleep, as well as the impact of worry. The impact of psychotic symptoms and of worry was also reported by participants in Waite et al. [38]. Those with post-partum psychosis reported the impact of worry regarding the wellbeing of their new-born [40], and the impact of feeling anxious and distressed, or of feeling elated, on sleep [52].

#### Perceived impact of psychiatric and psychological factors–quantitative evidence

Two quantitative studies showed similar findings regarding the perceived impact of mental health and of worry. Chiu et al. [53], found amongst inpatients with psychotic illnesses the most endorsed causes of sleep problems included ‘your illness’ (76%), worries and negative thoughts (72%), stress (72%), and medications (64%). Compared to community controls with insomnia participants with psychosis were ten times more likely to endorse certain biological causes (medication, illness, ‘problems with the brain’), and they were also less likely to see some lifestyle factors as affecting sleep, especially irregular sleep/wake schedule (p = 0.003). Sobieraj et al. [42], using a questionnaire with psychiatric outpatients, reported some similar findings, participants felt their sleep problem had occurred due to three potentially biological causes: since developing a particular condition (25%) for as long as they remembered (23%) or since taking a particular medication (15%). The most commonly rated cause was that sleep problems occurred ‘since a particular event’ (38%), however this is uninformative as it appears this could have been selected in relation to a life event, or a health event.

#### Acknowledgment of multiple causes of sleep problems

Caution should be applied in interpreting Sobieraj et al.’s [42] results as it appears participants were forced to choose a single cause, whilst when given the option participants selected multiple contributing factors [53]. Collier et al.’s qualitative study [41] also confirms that participants with mixed SMI perceived insomnia as interlinked with *multiple* other factors.

#### Perceived impact of environmental factors in acute hospitals

Both post-partum psychosis studies mentioned the negative impact of sleep loss from environmental causes; chiefly being woken by staff to breastfeed in the period immediately following labour, when participants felt they needed sleep to recover [40,52]. Similarly in inpatient mental health participants described the ward environment blighting their self-help attempts, through rules such as being prevented from making herbal tea at night [41]. These findings are corroborated in Chiu et al.’s quantitative study [53], as inpatients with psychosis often rated the environment as a cause of sleep problems, more-so than those with insomnia in the community.

#### Studies in bipolar

In contrast to findings in participants with psychotic illness above [53], findings in two studies in bipolar suggested sleep timing was seen as very important. Harvey et al. [54] found using quantitative methods, that euthymic patients with bipolar more frequently reported that sleep difficulties were due to being ‘unable to get into a proper routine’, than did those with insomnia without co-morbidity. This finding was corroborated by qualitative research: in Samalin et al. [55] participants with bipolar elaborated that they were oversensitive to any mis-timed zeitgebers, or departures from their normal routine, which would throw their sleep pattern out for days. The agreement between studies of differing methods adds weight to this finding.

#### Summary

People with SMI are aware of a range of psychiatric, lifestyle and environmental factors impacting on sleep. The impact of mental health factors on sleep was agreed upon, and received most coverage, environmental factors were also recognised, whilst other lifestyle factors received limited coverage. There were for instance no reports regarding the perceived impact of employment, or home environment. Studies in bipolar suggest a perception of an increased sensitivity to the effect of environmental cues on sleep timing, a finding which was not common to studies in other diagnostic groups.

### 4) Experiences of others’ attitudes toward sleep problems

In Engqvist and Nilsson's [40] study on postpartum psychosis it was noted that next of kin and staff had not initially reacted when they observed the womens’ loss of sleep, it was suggested that participants felt that the loss of sleep should have been reacted to, and was neglected. Similarly participants with mixed SMI in Collier et al. felt their sleep problems were misunderstood or neglected by staff, linking this to conflict and poor communication [41]. In contrast to this perceived neglect of sleep problems, euthymic patients with bipolar, felt that psychiatrists over prioritised discussion of sleep as a relapse indicator, while residual symptoms in the areas of social and cognitive functioning remained neglected [55].

The above studies discussed differences in the perceived importance of sleep between stakeholders. By contrast MacDonald et al. [37] described what they felt was an unexpected consensus between service user and psychiatrist participants, when they were interviewed together, regarding the importance of sleep problems, and the difficulties of managing them. No quantitative studies asked about participant’s experiences of other’s attitudes. It is likely that people with SMI’s experiences of others attitudes toward sleep problems will vary between groups and treatment contexts, but the current evidence base was not sufficiently developed to elaborate further regarding this.

### 5) Perspectives on approaches to improve sleep

#### Sleep hygiene and sleep education

Chiu et al. [53] quantitatively surveyed participants’ knowledge regarding sleep hygiene measures (and elicited their beliefs regarding the effect of these measures). Inpatients with psychotic illnesses and sleep problems endorsed less knowledge of sleep hygiene practices than did community controls. In particular they were less likely to endorse the negative effect of irregular sleep schedule, or of exercise directly before bed. They did however report better knowledge of the effect of caffeine on sleep than community controls with insomnia.

MacDonald et al.’s small scale qualitative study (n = 6 participants with SMI) echoes the suggestion of both limited knowledge of, and belief in, the effect of sleep hygiene measures [37]. Similarly Waite et al.’s participant’s with psychosis described sleep hygiene reading as not particularly beneficial [38]. By contrast participants in Waters et al. [56] were noted to be well informed regarding practical self-help solutions (‘sleep hygiene’ advice) and reported using these. In Holmes et al. [51] participants with mixed SMI appeared to be equally aware of self-help strategies, and they suggested these as relevant contents for a course to improve sleep. It is unclear however whether suggestions were a reflection of what participants felt worked for them personally, or whether participants reported ideas they knew were ‘supposed to work’.

Two studies surveyed perceived needs for health education in participants with schizophrenia, education regarding ‘sleeping problems’ was ranked as middling [57], or low in importance [58]. This contrasts to findings above, in depression and in psychotic illnesses, where sleep problems were seen as relatively important [46–48]. The importance patients assigned to sleep education may reflect the extent to which they want good sleep, the extent to which they perceive their sleep to be inadequate, their views of the effectiveness of sleep education, or their appraisal of their existing knowledge of sleep. Patients’ reasons for their ratings was not elicited in these studies.

#### Hypnotics

Peacey et al. [59] surveyed participants with mixed SMI, finding that they saw hypnotics as effective but wanted to discontinue their use because of adverse effects, they felt hypnotic use was unnatural, and not a long-term solution. Most disagreed that medication was the only solution to sleeplessness. But despite these reservations they still felt it was preferable to take hypnotics than to have a poor night’s sleep.

Limited qualitative evidence suggested some agreement regarding this fear or dislike of hypnotics, despite perceived efficacy. MacDonald et al.’s participants with mixed SMI described long term hypnotic use as a harsh necessity, preferring a natural solution, but not having faith in the efficacy of these [37]. Expressing similar concerns but a different conclusion, participants with psychotic illnesses in Waters et al. [56] described pharmacotherapy as effective, but unacceptable due to adverse effects, and also a lack of control over the process. Collier et al. [41] echoed this feeling of lack of control with use of hypnotics, but specifically expressed frustration with conflicting information about timings at which hypnotics could be given in inpatient settings.

#### Melatonin

In Waters et al. [56] some felt melatonin was more natural than hypnotics, and thus preferable. This was the only study describing perspectives on melatonin treatment.

#### CBT-i

Most participants in Waters et al. [56] felt CBT-type approaches were most preferable, based on their evaluation of CBT-i as described in a leaflet. Participants with psychosis in Waite et al. [38] admitted having varied expectations regarding efficacy of CBT-i before treatment. Following CBT-i participants were very positive regarding perceived efficacy, lack of side effects, and the collaborative nature of the treatment experience, although some had reservations about ‘too much talking’. This was the only study which qualitatively evaluated a well described treatment’s acceptability as part of an efficacy trial.

#### Challenges in the treatment of sleep problems

Many participants with psychosis noted that the appropriate solution to a sleep problem was highly individual and context dependent [56], in-keepingly Waite et al.’s highly individualised adaptation of CBT-i was positively evaluated in this population [38]. Both patients and psychiatrists agreed that sleep problems were complex, and that limited time during consultations was a barrier to their adequate exploration [37], whilst exploring causes of problems, developing understanding, and sharing treatment rationales was valued [38].

A range of outlooks were described, from seeing sleep as challenging but able to be improved, to feeling helpless. The inability to sleep felt uncontrollable to those developing post-partum psychosis [40], some participants felt helpless and hopeless regarding a solution [52], and a significant minority tried nothing to resolve their sleep problems [42]. Overcoming sleep problems through non-pharmacological approaches was seen as presenting significant personal challenges [38,56], taking time, effort and planning [37,38] and requiring effort and self-control [39]. Some felt resigned that sleep problems were inevitable, although many expressed hope for improvement.

## Discussion

This review highlights the strikingly limited number and quality of studies exploring or measuring the subjective perspectives of people with SMI regarding their sleep. There were only eleven studies which focused on this topic. Although many studies examine sleep in people with SMI, through objective measurement, there were very few which focused on patient perspectives. The movement for co-production and service user involvement in health service design [60,61] underlines the importance of the inclusion of service user perspectives at an early stage in the development of interventions, therefore it is important that these views are sought. Rigorous qualitative designs are needed which explore the experience and nature of sleep disturbance in different diagnostic groups and contexts, these studies should articulate patient priorities for treatment. Further studies comparing patient perspectives to staff perspectives may also be useful.

There is a drive to reduce inequality between physical health care and mental health care [62], research on perspectives on sleep problems in SMI must therefore catch up somewhat with research on sleep problems in people without SMI. For comparison, whilst this review retrieved eleven studies focused on this topic, recent reviews of patient perspectives on insomnia and its treatment, retrieved forty-three [63] and fifty-seven [64] studies respectively. The former [63] includes one article in common with the current review [41], whose sample was mixed SMI, whilst the latter [64] contains none in common. Few studies in either review included participants with SMI. Although these findings clearly have relevance, given the recognised importance of personalised healthcare [65], it is not sufficient to rely solely on the evidence base of general sleep medicine.

Sleep disturbance in SMI remains unduly neglected, covered slightly by the field of sleep medicine, and slightly by the field of mental health and psychiatry, but as yet, adequately covered by neither. Perhaps because of this sleep remains similarly neglected in clinical guidance; although sleep is discussed in guidance regarding depression [66], it is notably absent from guidance for bipolar disorder [67], for psychosis with coexisting substance misuse [68], and for psychosis and schizophrenia [69]. The latter suggests monitoring for co-existing conditions, and assessment of physical, social, and occupational factors [69], however despite their prevalence, sleep problems are not amongst the factors explicitly listed. It is not clear whether sleep received limited attention in guidance due to a lack of attention in practice, or vice versa.

Sleep disturbance is a common experience in people with SMI, yet there were only two studies which set out to qualitatively explore this experience [38,41]. Three further qualitative studies focused only on views regarding interventions [37,51,56], and the others were not focused on sleep [39,40,52,55]. There were no qualitative studies at all covering any aspect of sleep related perspectives or experiences in populations with severe depression, or with personality disorder. Qualitative studies are needed which explore the subjective experience of different types of sleep problem, in different diagnostic groups and clinical settings. These would better inform the development and adaptation of models of the mechanisms causing and perpetuating sleep disturbance, and could support development of more acceptable treatments, and better treatment compliance.

A few of the included quantitative studies suggested sleep expectations, priorities and beliefs in people with SMI may differ from those of healthy controls, and those with insomnia without co-morbidity, but evidence was limited. Furthermore diverse methodologies and terminology made interpretation difficult. More evidence is needed regarding sleep expectations and priorities in people with SMI. Further quantitative and mixed methods studies comparing objectively measured sleep, with sleep satisfaction, would be useful to examine what drives the perception of a sleep problem, and what drives presentation requesting treatment.

Qualitative exploration is also needed, which explores subjective evaluations of acceptability, desirability, and relative importance of various aspects of sleep, without reliance on expected norms. This could help to inform when intervention might be desired; who presents for treatment, and why some might not seek treatment even though they would benefit. This would also clarify which outcomes are important to people, and therefore which should be measured in efficacy studies. The current findings suggest existing outcome measures may require adaptation, however further exploration of patient priorities is required before more specific recommendations can be made.

There was some consensus that sleep was viewed as a complex area to address, and that participants perceived the bi-directional relationships between sleep and mental state. There was a little evidence suggesting more frequent attribution of sleep problems (rightly or wrongly) to biological causes, in people with SMI. Further exploration of sleep beliefs, and their relationships with subjective and objective sleep disturbance in people with SMI may be merited, both through qualitative exploration, and through correlational analysis. Perceptions regarding causes may impact on development or maintenance of a sleep problem, and on adherence to recommendations, advice, and treatment, as behaviour is known to be influenced by expected consequences.

There was minimal qualitative data, and even less quantitative data, regarding people with SMI’s pre-treatment perceptions of sleep interventions. Studies agreed that something *natural* is in principle preferable, but differing views regarding effectiveness of non-pharmacological approaches leads to expression of differing treatment preferences. There were some agreement regarding negative perceptions of sleep education and sleep hygiene, however the reasons for these were poorly explored or examined by qualitative or quantitative studies; this merits future attention. Better knowledge of pre-treatment perceptions might improve how interventions are presented and introduced.

Waite et al. [38] represents the first example of satisfaction data of any format collected during an effectiveness study. Further satisfaction data should be collected in future trials of treatments. Data collected as part of a trial may prove particularly useful, as treatments going by the same name can vary widely, and in these embedded studies a detailed account of the evaluated treatment should be available. Pre-treatment expectations and preferences may differ significantly from retrospective evaluations, and these might then be fruitfully compared.

### Limitations

It is acknowledged that some effectiveness studies may have included evaluations of user’s perspectives which were not mentioned in the abstract, and these may not have been identified. However inclusion of methodological design terms in the search strategy raises confidence that any such nested studies would have been picked up.

This review has highlighted the lack of evidence regarding perspectives on sleep in people with SMI, in order to do so all studies which might have addressed this topic were included. However this has meant bringing together evidence from diverse populations and settings, with diverse methodologies, measures and aims. Once a larger quantity of relevant evidence has accrued a more focused review, examining a single diagnosis, or a more focused part of this topic, might be beneficial. The current diverse inclusions made synthesis and comparison difficult, and reduces the ability to draw firm conclusions. The findings presented are therefore tentative, often supported by few studies. Furthermore limited description of the samples in some studies increases uncertainty regarding generalisability of some findings. It is recommended that future studies give clear descriptions of their samples, separating findings for sub-groups where possible, to allow future synthesis.

A further difficulty in completing this review was that although many measures included items indicative of relevant attitudes or beliefs, these were often embedded within a summary score, preventing some relevant comparisons being made. It is a further recommendation of this review that future studies using standardised scales such as the DBAS and PSQI, present itemised scores to enable more fruitful synthesis or meta-analysis of their findings.

## Conclusion

Although sleep disturbance is highly prevalent in people with SMI, relatively little is known about the experiences, priorities, and treatment preferences of these populations. It is important that the perspectives and experiences of people with SMI, regarding sleep, do not remain neglected. These perspectives form a crucial foundation to support the development and adaptation of interventions to improve sleep, and thereby improve mental and physical health, in these populations.

## Supporting Information

S1 File Critical Appraisal Criteria Used. (DOCX)

S2 File PRISMA Checklist. (DOC)

S3 File Articles excluded after seeking full text. (DOCX)

S1 Table Medline Search. (DOCX)

S2 Table Study Overviews. (DOCX)

S3 Table Critical Appraisal Summaries. (DOCX)
