# Supplementary material for: Perspectives on Sleep, Sleep Problems, and Their Treatment, in People with Serious Mental Illnesses: A Systematic Review
Source: PLoS One. 2016 Sep 22;11(9):e0163486. doi: 10.1371/journal.pone.0163486 (PMC5033349; doi:10.1371/journal.pone.0163486)
Supplement: S1 File — (DOCX) [file pone.0163486.s001.docx]

| **Appraisal prompts for qualitative studies** | |
| --- | --- |
| **Sample** | Was the sample used in the study appropriate to its research question? |
| **Data collection** | Were the data collected appropriately? |
| **Qualitative analysis** | Were the data analysed appropriately? |
| **Ethics and reflexivity** | Does the study adequately address potential ethical issues which could impact on the findings, including reflexivity?  For example coercion may affect the views people give, their openness or defensiveness, whilst participant debriefing occurs after data collection and so does not affect interpretation of the study findings. |
| Consider in relation to all of the above: | Is what the researchers did clear?  If methods are not described transparently this should raise suspicions regarding quality. |
| Generalisability | To what extent can we transfer the results of this study to people with SMI in general? |

Prompts adapted from Kuper et al. (2008), guidance from Greenhalgh (2010) was also referred to in answering these questions.

| **Appraisal prompts for quantitative studies** | |
| --- | --- |
| **Sample** | Appropriate sampling strategy, sample size, representativeness, exclusions, response rate, reasons for non-participation, withdrawal, missing data. |
| **Validity and reliability of measures** | Conceptual congruence, patient relevance, construct validity, concurrent validity if applicable, reliability - *in the population studied*. |
| **Administration** | When, where, by whom, factors relating to use of repeated measures, consistent use between sites / groups, length and number of measures used – burden, other potential sources of bias. |
| **Analysis and interpretation** | Appropriateness of comparisons where present, appropriate statistical methods, identification of clinical significance of change / differences, inferences consistent with the strength of the data, alternative explanations. |
| Generalisability | To what extent can we generalise the results of this study to people with SMI? |

Questions and prompts adapted from the recommendations of Greenhalgh (2010), Acaster et al. (2012) and Mokkink et al. (2010).

Appraisals are summarised as POOR, MEDIUM, GOOD or UNCLEAR in S3_Tables. Assessments of generalisability were not summarised in this way as this would not be meaningful without additional explanation.
