# Supplementary material for: Perspectives on Sleep, Sleep Problems, and Their Treatment, in People with Serious Mental Illnesses: A Systematic Review
Source: PLoS One. 2016 Sep 22;11(9):e0163486. doi: 10.1371/journal.pone.0163486 (PMC5033349; doi:10.1371/journal.pone.0163486)
Supplement: S3 File — (DOCX) [file pone.0163486.s003.docx]

| **Reason for exclusion** | **Number of articles excluded** |
| --- | --- |
| Unable to obtain full text | 1 |
| Not available in English | 1 |
| Did not meet population inclusion criteria | 14 |
| May mention but does not discuss sleep | 11 |
| No patient attitudes, beliefs or perspectives relating to sleep or sleep treatment presented | 36 |

**Unable to obtain full text**

1. Primerano G, Casali F. Psychoeducational activity in the treatment of insomia in a psychiatric inpatient facility. <L’intervento psicoeducativo nel trattamento dell'insonnia in una struttura psichiatrica residenziale.>. Psicoter Cogn e Comport. 2006;12(2):203–16.

**Not available in English**

1. Voinescu BI, Coogan AN, Orasan R. Sleep beliefs, subjective sleep quality and diurnal preference - Findings from depressed patients. J Cogn Behav Psychother. 2010;10(1):1–12.

**Did not meet population inclusion criteria:**

1. Brand S, Gerber M, Puhse U, Holsboer-Trachsler E. Depression, hypomania, and dysfunctional sleep-related cognitions as mediators between stress and insomnia: The best advice is not always found on the pillow! International Journal of Stress Management. 2010. p. 114–34.

2. DeViva JC, Zayfert C, Pigeon WR, Mellman TA. Treatment of Residual Insomnia After CBT for PTSD: Case Studies. J Trauma Stress. 2005;18(2):155–9.

3. Engdahl BE, Eberly RE, Hurwitz TD, Mahowald MW, Blake J. Sleep in a community sample of elderly war veterans with and without posttraumatic stress disorder. Biol Psychiatry. 2000;47(6):520–5.

4. Edinger JD, Olsen MK, Stechuchak KM, Means MK, Lineberger MD, Kirby A, et al. Cognitive behavioral therapy for patients with primary insomnia or insomnia associated predominantly with mixed psychiatric disorders: A randomized clinical trial. Sleep. 2009;32(4):499–510.

5. Fairholme C, Manber R. Safety behaviors and sleep effort predict sleep disturbance and fatigue in an outpatient sample with anxiety and depressive disorders. J Psychosom Res. 2014;76(3):233–6.

6. Gellis LA, Gehrman PR. Cognitive behavioral treatment for insomnia in veterans with long-standing posttraumatic stress disorder: A pilot study. J Aggress Maltreat Trauma. 2011;20(8):904–16.

7. Hsu H-M, Chou K-R, Lin K-C, Chen K-Y, Su S-F, Chung M-H. Effects of cognitive behavioral therapy in patients with depressive disorder and comorbid insomnia: A propensity score-matched outcome study. Behav Res Ther. 2015;73:143–50.

8. Klein E, Koren D, Arnon I, Lavie P. Sleep complaints are not corroborated by objective sleep measures in post-traumatic stress disorder: a 1-year prospective study in survivors of motor vehicle crashes. J Sleep Res. 2003;12(1):35–41.

9. Krakow B, Johnston L, Melendrez D, Hollifield M, Warner TD, Chavez-Kennedy D, et al. An open-label trial of evidence-based cognitive behavior therapy for nightmares and insomnia in crime victims with PTSD. Am J Psychiatry. 2001;158(12):2043–7.

10. Koren D, Arnon I, Lavie P, Klein E. Sleep Complaints as Early Predictors of Posttraumatic Stress Disorder: A 1-Year Prospective Study of Injured Survivors of Motor Vehicle Accidents. Am J Psychiatry. 2002;159:855–7.

11. Lichstein KL, Wilson NM, Johnson CT. Psychological Treatment of Secondary Insomnia. Psychol Aging. 2000;15(2):232–40.

12. Magnúsdóttir SD, Ribacke M. Patients’ knowledge and attitudes regarding sleep and hypnotics. Scand J Prim Health Care. 1996;14(2):106–10.

13. Russinova Z, Cash D, Wewiorski NJ. Toward Understanding the Usefulness of Complementary and Alternative Medicine for Individuals With Serious Mental Illnesses Classification of Perceived Benefits. J Nerv Ment Dis. 2009;197(1):69–73.

14. Tsai Y-L, Chen C-W, Cheng H-C, Chang C-H, Chen C-Y, Yang C-M. Cognitive and behavioral factors in insomnia comorbid with depression and anxiety. Sleep Biol Rhythms. 2013;11(4):237–44.

**May mention but does not discuss sleep:**

1. Solomon P, Beck S. Patients’ perceived service needs when seen in a psychiatric emergency room. Psychiatr Q. 1989;60(3):215–26.

2. Chernomas WM, Clarke DE, Chisholm F a. Perspectives of women living with schizophrenia. Psychiatr Serv. 2000;51(12):1517–21.

3. Comtois G, Morin C, Lesage A, Lalonde P, Likavcanova E, L’Écuyer G. Patients Versus Rehabilitation Practitioners: A Comparison of Assessments of Needs for Care. Can J Psychiatry. 1998;43:159–65.

4. Heron J, Gilbert N, Dolman C, Shah S, Beare I, Dearden S, et al. Information and support needs during recovery from postpartum psychosis. Arch Womens Ment Health. 2012;15(3):155–65.

5. Faulkner G, Sparkes A. Exercise as therapy for schizophrenia: an ethnographic study. Journal of Sport and Exercise Psychology. 1999. p. 52–69.

6. Karanci AN. Causal attributions for illness among Turkish psychiatric out-patients and differences between diagnostic groups. Social Psychiatry and Psychiatric Epidemiology. Germany: Springer; 1993. p. 292–5.

7. Lynch MM, Kruzich JM. Needs assessment of the chronically mentally ill: Practitioners and client perspectives. Adm Ment Health. 1986;13(4):237–48.

8. Minato M, Zemke R. Time use of people with schizophrenia living in the community. Occup Ther Int. 2004 Aug;11(3):177–91.

9. Noordsy DL, Drake RE, Teague GB, Osher FC, Hurlbut SC, Beaudett MS, et al. Subjective Experiences Related to Alcohol Use among Schizophrenics. J Nerv Ment Dis. 1991;179(7):410–4.

10. Ronan P, Robinson N, Harbinson D, Macinnes D. A case study exploration of the value of acupuncture as an adjunct treatment for patients diagnosed with schizophrenia: Results and future study design. J Chinese Integr Med. 2011;9(5):503–14.

11. Sheehan W, Kroll J. Psychiatric Patients’ Belief in General Health Factors and Sin as Causes of Illness. Am J Psychiatry. 1990;147:112–3.

**No patient attitudes, beliefs or perspectives relating to sleep or sleep treatment presented:**

1. Afonso P, Figueira ML, Paiva T. Sleep-promoting action of the endogenous melatonin in schizophrenia compared to healthy controls. Int J Psychiatry Clin Pract. 2011;15(4):311–5.

2. Afonso P, Canas F, Bobes J, Bernardo Fernandez I, Guzman C. Satisfaction with Life of Schizophrenia Outpatients and Their Caregivers: Differences between Patients with and without Self-Reported Sleep Complaints. Schizophr Res Treatment. 2013;

3. Afonso P, Brissos S, Cañas F, Bobes J, Bernardo-Fernandez I. Treatment adherence and quality of sleep in schizophrenia outpatients. Int J Psychiatry Clin Pract. 2014 Jan;18(1):70–6.

4. Chellappa SL, Araujo JF. Sleep disorders and suicidal ideation in patients with depressive disorder. Psychiatry Res. 2007;153:131–6.

5. Bloch B, Vadas L, Haliba Y. The Effects of Music Relaxation on Sleep Quality and Emotional Measures in People Living with Schizophrenia. J Music Ther. 2010;47(1):27–52.

6. Cui L, Ke-qing L, Xiuli S, Ze C, Qinpu J, Yanchao H, et al. A Survey of Sleep Quality in Patients With 13 Types of Mental Disorders. Prim Care Companion CNS Disord. 2012;14(6).

7. Dursun SM, Patel JKM, Burke JG, Reveley MA. Effects of Typical Antipsychotic Drugs and Risperidone on the Quality of Sleep in of Patients With Schizophrenia : a Pilot Study. J Psychiatry Neurosci. 1999;24(4):333–7.

8. Hofstetter JR, Mayeda AR, Happel CG, Lysaker PH. Sleep and Daily Activity Preferences in Schizophrenia : Associations with Neurocognition and Symptoms (brief report). J Nerv Ment Dis. 2003;191(6):408–16.

9. Schäfer V, Bader K. Relationship between early-life stress load and sleep in psychiatric outpatients: A sleep diary and actigraphy study. Stress Heal. 2013 Aug;29(3):177–89.

10. Hamera E, Brown C, Goetz J. Objective and subjective sleep disturbances in individuals with psychiatric disabilities. Issues Ment Health Nurs. 2013 Feb;34:110–6.

11. Hofstetter JR, Lysaker PH, Mayeda AR. Quality of sleep in patients with schizophrenia is associated with quality of life and coping. BMC Psychiatry. 2005;5(13).

12. Kallestad H, Hansen B, Langsrud K, Ruud T, Morken G, Stiles TC, et al. Differences between patients’ and clinicians' report of sleep disturbance: a field study in mental health care in Norway. BMC Psychiatry. 2011;11(186).

13. Kodaka M, Matsumoto T, Katsumata Y, Akazawa M, Tachimori H, Kawakami N, et al. Suicide risk among individuals with sleep disturbances in Japan: a case-control psychological autopsy study. Sleep Med. 2014 Apr;15(4):430–5.

14. Kohn L, Espie C a. Sensitivity and specificity of measures of the insomnia experience: a comparative study of psychophysiologic insomnia, insomnia associated with mental disorder and good sleepers. Sleep. 2005;28(1):104–12.

15. Krahn L, Lin S, Wisbey J, Rummans T, O’Connor M. Assessing sleep in psychiatric inpatients: nurse and patient reports versus wrist actigraphy. Ann Clin Psychiatry. 1997;9(4):203–10.

16. Kumar PNS, Andrade C, Bhakta SG, Singh NM. Melatonin in schizophrenic outpatients with insomnia: A double-blind, placebo-controlled study. J Clin Psychiatry. 2007;68(2):237–41.

17. Laguna-Parras JM, Jerez-Rojas MR, Garcia-Fernandez FP, Carrasco-Rodriguez MD, Nogales-Vargas-Machuca I. Effectiveness of the “sleep enhancement” nursing intervention in hospitalized mental health patients. J Adv Nurs. 2013;69(6):1279–88.

18. Lusignan F-A, Zadra A, Dubuc M-J, Daoust A-M, Mottard J-P, Godbout R. Dream content in chronically-treated persons with schizophrenia. Schizophr Res. 2009;112(1-3):164–73.

19. Mayers AG, van Hooff JC, Baldwin DS. Quantifying subjective assessment of sleep and life-quality in antidepressant-treated depressed patients. Hum Psychopharmacol. 2003;18:21–7.

20. McCall W V, Blocker JN, D’Agostino Jr R, Kimball J, Boggs N, Lasater B, et al. Treatment of insomnia in depressed insomniacs: effects on health-related quality of life, objective and self-reported sleep, and depression. J Clin Sleep Med. 2010;6(4):322–9.

21. Reshef A, Bloch B, Vadas L, Ravid S, Kremer I, Haimov I. The effects of acupuncture treatment on sleep quality and on emotional measures among individuals living with schizophrenia. Sleep Disord. 2013;14:e147–8.

22. Semiz UB, Basoglu C, Ebrinc S, Cetin M. Nightmare disorder, dream anxiety, and subjective sleep quality in patients with borderline personality disorder. Psychiatry Clin Neurosci. 2008;62(1):48–55.

23. Shamir E, Laudon M, Barak Y, Anis Y, Rotenberg V, Elizur A, et al. Melatonin Inproves Sleep Quality of Patients With Chronic Schizophrenia. J Clin Psychiatry. 2000;61(5):373–7.

24. Sugishita K, Yamasue H, Kasai K. Continuous positive airway pressure for obstructive sleep apnea improved negative symptoms in a patient with schizophrenia. Psychiatry Clin Neurosci. 2010;64(6):665.

25. Thompson L, Pennay A, Zimmermann A, Cox M, Lubman DI. “Clozapine makes me quite drowsy, so when I wake up in the morning those first cups of coffee are really handy”: an exploratory qualitative study of excessive caffeine consumption among individuals with schizophrenia. BMC Psychiatry. 2014;14(1):116.

26. Waters F, Faulkner D, Naik N, Rock D. Effects of polypharmacy on sleep in psychiatric inpatients. Schizophr Res. 2012;139(1-3):225–8.

27. Waters F, Naik N, Rock D. Sleep, fatigue, and functional health in psychotic patients. Schizophr Res Treatment. 2013;2013.

28. Wulff K, Joyce E, Middleton B, Dijk D-J, Foster RG. The suitability of actigraphy, diary data, and urinary melatonin profiles for quantitative assessment of sleep disturbances in schizophrenia: A case report. Chronobiol Int. 2006;23(1&2):485–95.

29. Argyropoulos S V, Hicks JA, Nash JR, Bell CJ, Rich AS, Nutt DJ, et al. Correlation of subjective and objective sleep measurements at different stages of the treatment of depression. Psychiatry Res. 2003;120(2):179–90.

30. Carney CE, Edinger JD, Manber R, Garson C, Segal Z V. Beliefs about sleep in disorders characterized by sleep and mood disturbance. J Psychosom Res. 2007;62(2):179–88.

31. Leufstadius C, LK E, Eklund M. Time use and daily activities in people with persistent mental illness. Occupational Therapy International. 2006. p. 123–41.

32. Eklund M, Erlandsson L, Leufstadius C. Time use in relation to valued and satisfying occupations among people with persistent mental illness: Exploring occupational balance. J Occup Sci. 2010;17(4):231–8.

33. Bastien CH, Guimond S, St-Jean G, Lemelin S. Signs of insomnia in borderline personality disorder individuals. J Clin Sleep Med. 2008;4(5):462–70.

34. Rotenberg V, Indurski P, Kimhi R, Hadjez J, Gutman Y, Shamir E, et al. The relationship between objective sleep variables and subjective sleep estimation in schizophrenia. Int J Psychiatry Clin Pract. 2000;4(1):63–7.

35. Boudebesse C, Geoffroy PA, Bellivier F, Henry C, Folkard S, Leboyer M, et al. Correlations between objective and subjective sleep and circadian markers in remitted patients with bipolar disorder. Chronobiol Int. 2014;31(5):698–704.

36. Michels F, Schilling C, Rausch F, Eifler S, Zink M, Meyer-Lindenberg A, et al. Nightmare frequency in schizophrenic patients, healthy relatives of schizophrenic patients, patients at high risk states for psychosis, and healthy controls. Int J Dream Res. 2014;7(1):9–13.
