# Supplementary material for: Perspectives on Sleep, Sleep Problems, and Their Treatment, in People with Serious Mental Illnesses: A Systematic Review
Source: PLoS One. 2016 Sep 22;11(9):e0163486. doi: 10.1371/journal.pone.0163486 (PMC5033349; doi:10.1371/journal.pone.0163486)
Supplement: S1 Table — (DOCX) [file pone.0163486.s004.docx]

|  | **Search terms** | **Results** |
| --- | --- | --- |
| 1 | sleep.hw. or Circadian Rhythm/ | 158,374 |
| 2 | (sleep or $somnia or circadian).ab. | 128,191 |
| 3 | 1 or 2 | 202,296 |
| 4 | *Mental Disorders/ or *psychotic disorders/ or *schizophrenia/ or *delusions/ or *Bipolar Disorder/ or *Depression/ or *Mood Disorders/ or *Personality Disorders/ or *Mental Disorders/ or *Depressive Disorder/ | 314,142 |
| 5 | (psychosis or psychotic or delusional or schizo* or psychiatric or (mental adj1 health) or (mental adj1 illness) or bipolar or bi-polar or manic or (affective adj1 disorder*) or depress* or (personality adj1 disorder)).ti. | 321,927 |
| 6 | 4 or 5 | 438,535 |
| 7 | *qualitative research/ or *Focus Groups/ or *Anthropology, Cultural/ or *Anthropology/ or *health impact assessment/ or *health surveys/ or *health care surveys/ or *questionnaires/ or *self report/ or *Personal Satisfaction/ | 60,090 |
| 8 | (attitude* or opinion* or belief* or view* or value* or perspective* or priorit* or prefer* or qualitative or interview or (focus adj1 group) or phenomeno* or perception* or perceived or self-report* or (self adj1 report) or user-rated or (user adj1 rated) or patient-rated or (patient adj1 rated) or questionnaire or survey or Likert or satisfaction).ti. | 646,255 |
| 9 | 7 or 8 | 679,642 |
| 10 | 3 and 6 and 9 | 476 |
| 11 | limit 10 to (English language and humans) | 380 |
| 12 | remove duplicates from 11 | 362 |
