# Supplementary material for: Perspectives on Sleep, Sleep Problems, and Their Treatment, in People with Serious Mental Illnesses: A Systematic Review
Source: PLoS One. 2016 Sep 22;11(9):e0163486. doi: 10.1371/journal.pone.0163486 (PMC5033349; doi:10.1371/journal.pone.0163486)
Supplement: S2 Table — (DOCX) [file pone.0163486.s005.docx]

| **Study Overviews: Qualitative Studies A-Z** | | | | |
| --- | --- | --- | --- | --- |
| **Reference**  *Short title*  (Country) | **Participants and setting** | **Aims and objectives** | **Research methods** | **Relevant findings reported** |
| **Collier et al. 2003**  *Insomnia in a psychiatric inpatient population*  (UK) | 7 psychiatric inpatients, admitted for at least a week.  Complaining of insomnia, demographics and medications given, psychiatric diagnosis not given.  57% male. | To identify the subjective experience of insomnia and its assessment and treatment in a  psychiatric inpatient population. | Semi-structured interviews. Analysed using a content analysis  framework modified from Burnard (1991). Information from staff report and notes was also taken into account. | -Insomnia seen as interlinked with other factors especially mental health  - Participants felt staff sometimes did not understand their experience and needs. There was a suggestion that sleep problems were not discussed with staff.  -Frustration with ward policies and conflicting messages especially re: timing and availability of hypnotics.  - Personal strategies included preparation and bedtime routine, relaxation received a mixed response.  -Hopes for the future, wish for normality, resignation. |
| **Davis & O’Neill 2005**  *Relapse prevention strategies in substance misuse*  (USA) | 27 outpatients, later stages of substance abuse treatment.  74% schizophrenia spectrum, 22% major affective disorder.  56% male. | To identify the strategies people with dual diagnosis rely on in avoiding relapse. | Focus groups of between 4 and 9 participants, thematic analysis | -Many participants described prioritising and normalising their sleep, as an important part of their now much improved personal care and lifestyle balance. There may have been some difficulty and effort involved, indicated by the description of ‘forcing yourself to sleep’. |
| **Engqvist et al. 2011**  *Postpartum psychotic episodes*  (International) | 10 online narratives (outpatients / retrospective).  Postpartum psychosis.  0% male. | To describe the womens’ perceived experience of post-partum psychosis. | Data sourced from online narratives. Cross-case analysis and content analysis was used, this was completed by a team, coding was used and themes developed. | -All but one narrative included a loss of sleep, women felt restless, distressed, or elated. The women couldn’t sleep, or were eventually given sleeping tablets and slept.  -Women were prevented from sleeping after delivery by interruptions to care for the baby, and felt disillusioned, helpless and poorly understood. |
| **Engqvist & Nilsson 2013**  *The first days of postpartum psychosis*  (Sweden) | 7 recovered participants.  Postpartum psychosis, 7 to 32 years since their illness.  0% male  Also: 6 next of kin | To explore experience of first days of postpartum psychosis from the womens’ and the next of kins’ perspectives. | Interviews, content analysis, coded for manifest and latent content. Themes compared to the data until coherent and complete. | -First theme: ‘Loss of sleep’ – unexpected, uncontrollable  -Difficulties sleeping caused by: ward environment, physical issues after giving birth, anxieties about checking on the baby, and staff waking women to breastfeed.  -Loss of sleep was described by some as though causal of subsequent deterioration in mental state.  -Next of kin and staff did not really react to loss of sleep. |

| **Study Overviews: Qualitative Studies A-Z (continued)** | | | | | | | | | |
| --- | --- | --- | --- | --- | --- | --- | --- | --- | --- |
| **Reference**  *Short title*  (Country) | | **Participants and setting** | | **Aims and objectives** | | **Research methods** | | **Relevant findings reported** | |
| **Holmes et al. 1995**  *Development of a sleep management program*  (USA) | | 23 participants from a partial hospitalisation programme.  53% schizophrenia, 13% bipolar disorder, 20% schizoaffective disorder, 6.7% major depression, and 6.7% other. Av. age 36.5  47% male  Comparison group: 22 college students, av. age 24. | | To develop a sleep training module, and to deliver and test it’s efficacy. Asked participants about problems relevant to their sleep, their coping responses, and what they feel an intervention should address. | | Mixed methods. Structured open ended questions, written responses to develop initial list. Participants then rated for relevance in later meetings. Training module tested by change in knowledge of sleep related facts, and self-reported restfulness of sleep. | | -Participant’s with SMI had more concerns about mental health related factors on sleep whilst college students had more concerns about daily stress and relationships. Both groups identified worry as affecting sleep.  -Participants with SMI were able to generate a range of solutions to problems, individually they offered less responses than controls, but as a group the same number of unique solutions were generated.  -The strategies of people with SMI were more likely to include contacting a healthcare worker or medication, and they suggested a wider range of practical strategies (note this could be because of their older age) | |
| **MacDonald et al 2015**  *‘Is it the crime of the century’: hypnosedative prescription*  (New Zealand) | | 6 outpatients.  Secondary care mental health, diagnoses not given, current or previous sleep problems.  33% male.  Also: 12 psychiatrists. | | To explore attitudes and motivations influencing long term hypnosedative use, and barriers to use of non-medication therapies. | | Focus groups (service users and psychiatrists separate, then later together), thematic analysis using data analysis software, and using multiple coders working separately then discussing amongst the team. | | -Service users and psychiatrists felt sleep problems were complex to manage, and felt there was insufficient time in consultations to explore sleep difficulties adequately.  -Non-pharmacological approaches were seen as requiring a lot of time, effort and knowledge.  -Service users were conflicted about taking hypnosedatives, describing them as a harsh necessity in the absence of effective alternatives.  -Service users lacked awareness of other approaches, and lacked faith in their effectiveness. | |
| **Samalin et al. 2014**  *Residual bipolar symptoms*  (France) | | 23 outpatients.  Bipolar I or II, have been euthymic for at least 6 months.  39% male. | | To explore patient’s perspectives on residual symptoms in bipolar. | | Seen in 3 focus groups of 6-11, thematic analysis, concurrent collection and analysis, large team coding, judged to be saturated after third focus group. | | -Residual sleep disturbances reported including problems with sleep latency, maintenance, and timing, and non-restorative sleep.  -Participants described oversensitivity to mistimed zeitgebers, or occasional departures from routine, throwing their routine out of synch.  -Participants felt reviewing psychiatrists were too focused on sleep, and that other issues were neglected including socio-professional and cognitive performance. | |
| **Study Overviews: Qualitative Studies A-Z (continued)** | | | | | | | | | |
| **Reference**  *Short title*  (Country) | | **Participants and setting** | | **Aims and objectives** | | **Research methods** | | **Relevant findings reported** | |
| **Waite et al. 2015**  *Sleep problems in people with psychosis*  (UK) | | 10 outpatients recruited from the active arm of a CBT-i trial (the Better Sleep Trial).  Schizophrenia, schizoaffective disorder or first episode psychosis.  30% male. | | To gain patient perspectives on the nature of sleep problems in psychosis and the experience of treatment (with individualised CBT-i). | | Semi-structured interviews, analysed using Interpretive Phenomenological Analysis, using independent coders and a reflexive log. Satisfaction also rated using a Likert scale. | | -Sleep problems were viewed as affecting emotions, causing fatigue, and impacting on participation in life.  -Participants noted a bidirectional relationship between psychotic experience, sleep disturbance and sleep habits.  -Following CBT-i participants described improved sleep and associated improvements in symptoms, functioning, self-esteem, lifestyle and life satisfaction.  -Participants varied in their expectations of efficacy but were willing to try CBT-i anyway.  -Participants appreciated the collaborative approach, and were mostly very satisfied. | |
| **Waters et al.**  **2015**  *Preferences for insomnia treatment in psychosis*  (Australia) | | 14 inpatients and outpatients recruited from a community drop-in centre.  Schizophrenia or schizoaffective disorder, all taking antipsychotics, ages 28-64.  50% male. | | To investigate the views and preferences of participants of 3 evidence based sleep interventions: 1 standard pharmacological, 2 melatonin, 3 psychological and behavioural type intervention. | | Semi-structured focus groups (x3, each up to 4 participants) and individual interviews (x6). Participants recruited via snowball sampling. Handout materials presented re: melatonin and CBT. Analysed using Krueger & Casey’s approach to sorting transcript data, multiple coders, proportions of participants endorsing different views were presented. | | -Pharmacotherapy was viewed as effective by some but as unacceptable by most.  -Melatonin was favoured because it was new, and perceived as more natural by some.  -CBT approaches were most favoured, because they were drug free, and promoted control and autonomy, whilst some were concerned about a lack of efficacy, and the discipline required to engage in these approaches.  -Many participants noted that different people may benefit from different approaches at different times.  -Engagement in CBT approaches benefitted from: hearing about outcomes, seeing improvement, peer support.  -Strategies reported were largely in line with those recommended by sleep organisations and the media. | |

| **Study Overviews: Quantitative Studies A-Z** | | | | |
| --- | --- | --- | --- | --- |
| **Reference**  *Short title*  (Country) | **Participants and setting** | **Aims and objectives** | **Research methods** | **Relevant findings reported** |
| **Auslander & Jeste 2002**  *Perceptions of problems and needs for service*  (USA) | 72 outpatients.  Schizophrenia and related psychotic disorders, middle aged and elderly, stable.  59% male. | To examine the perceptions of problems and needs for services. | Survey, based on items generated from 3 focus groups with patients (av. 15 per group) and 2 with healthcare providers (av. 5 per group). Items rated (Likert scales) and ranked. | “To sleep better” was ranked 6^th^ most important out of 50 items, and ranked above developing more friendships, improving mood, or being more comfortable around people. |
| **Chiu et al. 2015**  *Insomnia in psychosis*  (Australia) | 55 inpatients.  76% with schizophrenia spectrum disorders, 25 with insomnia  75% male.  Comparison group: 66 community controls, 25 with insomnia. | To examine which sleep-related cognitive and behavioural factors are associated with insomnia in people with or without a psychotic illness. | Cross-sectional comparison of results on measures including the DBAS, Sleep Hygiene Knowledge Scale, and Causes of sleep problems questionnaire. Analysed using inferential statistics. | DBAS scores were higher in both insomnia groups, in the psychosis group this was mediated by negative mood. Inpatients were less well informed re: sleep hygiene than controls, caffeine use and mood were well endorsed as important, whilst nicotine and daylight exposure were least well endorsed across all groups. Inpatients were more poorly informed regarding the effect of irregular sleep schedules than controls. Inpatients endorsed more biological causes of sleep problems, especially their illness as a cause, whilst controls more often endorsed lifestyle factors. |
| **Harvey et al. 2005**  *Sleep in bipolar disorder*  (UK and USA) | 20 outpatients.  Bipolar, currently euthymic.  50% male.  Comparison groups: 20 insomnia, 20 good sleepers. | To investigate sleep related functioning in euthymic patients with bi-polar | Comparison of data from interviews, questionnaires and actigraphy between groups. The insomnia diagnostic interview, PSQI, sleep diary, Sleep Disturbance Questionnaire and DBAS were used. | There were some minor differences in which PSQI subscales were scored highly in by people with bipolar and with insomnia. Those with bipolar reported more problems getting into a routine than people with insomnia. DBAS scores were similar between people with insomnia and bipolar. Sleep hygiene in both groups was no different to good sleepers. People with bipolar had lower activity levels in the day. |

| **Study Overviews: Quantitative Studies A-Z (continued)** | | | | |
| --- | --- | --- | --- | --- |
| **Reference**  *Short title*  (Country) | **Participants and setting** | **Aims and objectives** | **Research methods** | **Relevant findings reported** |
| **Li et al.**  **2011**  *Dysfunctional sleep beliefs in treatment of depression*  (China) | 66 inpatients.  Major depression (average Hamilton Depression Rating Scale score of 25.4).  44% male. | To explore what sleep beliefs contribute to sleep disturbance after depression and anxiety are treated. | Participants completed the DBAS-16, PSQI and Hamilton depression and anxiety rating scales. Participants were then treated for depression with alprazolam and paroxetine for four weeks and assessed again, comparing before and after measurements. | DBAS scores actually increased after successful pharmacological treatment of depression and anxiety, PSQI scores had not changed significantly. Participants had become significantly less anxious and depressed, but had become significantly more worried about insomnia, perceived more consequences of insomnia, and had greater ‘dysfunctional sleep expectations’. |
| **Lien et al. 2003**  *Health Education Needs of patients with schizophrenia*  (China) | N=?, inpatients.  Schizophrenia.  (further details sought without success).  Comparison group: 129 professionals. | To compare the perceptions of patient educational needs between patients and professionals. | Non-experimental descriptive survey study. | Patients ranked education about sleep as of low importance, whilst HCP’s especially medical HCPs ranked sleep as amongst the most important topics. |
| **Mueser et al. 1992**  *Educational needs of patients and relatives*  (USA) | 60 outpatients.  46 schizophrenia or schizoaffective disorder, 14 major affective disorder.  Also: 108 relatives and 27 close friends. | To determine the perceived educational needs of different groups of patients and their friends and relatives. | A questionnaire was developed with the help of 10 professionals, 5 family members and 5 patients. Likert scales to rate level of interest in the 45 listed topics. | Sleeping problems were rated as 41^st^ out of 45 topics for interestedness in education by patients with schizophrenia and schizoaffective disorder, and as 23^rd^ out of 45 by patients with major affective disorder. |
| **Niet de et al. 2008**  *Perceived sleep quality of psychiatric patients*  (Netherlands) | 560 inpatients and outpatients from 6 different psychiatric institutions.    Diagnoses not reported, mean age 56.1.  46% male. | To gain knowledge about quality of sleep in adult and elderly  psychiatric patients, and to identify key  factors in perceiving a sleep problem. | Cross sectional design, participants were asked whether they thought they had a sleep problem, and completed the PSQI. A logistic regression was performed to determine which PSQI subscales impacted on perception of a sleep problem. | The answers to some questions on the PSQI impact on the person’s appraisal of the adequacy of their sleep (keeping up enthusiasm to get things done, waking in the night, having bad dreams, trouble staying awake, sleep medication use) whilst other factors have little impact (bathroom use) suggesting they were not experienced as contributing toward a ‘sleep problem’. |

| **Study Overviews: Quantitative Studies A-Z (continued)** | | | | |
| --- | --- | --- | --- | --- |
| **Reference**  *Short title*  (Country) | **Participants and setting** | **Aims and objectives** | **Research methods** | **Relevant findings reported** |
| **Pandina et al. 2010**  *Patient-Rated Symptom Scale for Depression*  (USA) | 268 in/outpatients.  Treatment resistant major depressive disorder.  27% male. | To validate the Patient Rated Troubling Symptoms of Depression (PaRTS-D) self-report measure. | Secondary statistical analysis of data from a large scale clinical trial including correlational analysis and assessment of test-retest reliability. | *Reduced sleep* often ranks amongst the most troubling symptoms, but not as the single most troubling symptom. |
| **Peacey et al. 2012**  *Sleep medications in acute illness*  (New Zealand) | 35 inpatients and 65 outpatients receiving active treatment for acute or chronic psychiatric illness.  Mixed SMI, breakdown by conditions presented, age 19-63.  54% male | To examine prescribing patterns of hypnotics and psychotropics with hypnotic effects, to measure patient knowledge and perception of efficacy. | Structured interview, the DBAS, and information gathered from notes, descriptive statistics were presented for outcomes with non-normal data and a statistical test was used for one outcome. | In response to the two questions on the DBAS regarding medication use most preferred taking a sleeping pill to having a poor night’s sleep, but most disagree or strongly disagree that medication is the only solution to sleeplessness. Patients reported a number of perceived effects of medication on sleep, some couldn’t recall being able to sleep without medication. They considered hypnotics effective but wanted to discontinue them. |
| **Plante et al. 2013**  *Maladaptive sleep cognitions in borderline personality disorder (BPD)*  (USA) | 223 outpatients who had had previous admissions.  BPD, ages 18-35, no co-morbid psychotic disorder or bipolar.  19% male. | Determine whether there is a link between rates of recovery from BPD and scores on DBAS | Longitudinal study (using data from the most recent follow up wave, 16 years on), DBAS scores were not obtained during previous follow up waves. | Participants who were recovered had lower DBAS scores. ‘Consequences’, ‘Worry/Helplessness’ and ‘Medication’ scores were elevated in non-recovered participants. OSA was also more common in those who had not recovered. |
| **Poulin et al.**  **2010**  *Sleep habits in schizophrenia*  (Canada) | 150 outpatients.  Schizophrenia and schizoaffective disorder.  69% male  Comparison group: 80 healthy controls | Compare sleep characteristics between groups | Sleep habits questionnaire administered and results compared. | Sleep latency and sleep duration, were greater in patients than in controls, as was amount of time in bed, and napping, but sleep satisfaction was not significantly lower than in controls. |

| **Study Overviews: Quantitative Studies A-Z (continued)** | | | | |
| --- | --- | --- | --- | --- |
| **Reference**  *Short title*  (Country) | **Participants and setting** | **Aims and objectives** | **Research methods** | **Relevant findings reported** |
| **Sobieraj et al. 2013**  *Sleep abnormalities questionnaire*  (Poland) | 49 psychiatry outpatients.    No information regarding diagnoses, average age 44.  43% male. | To look at occurrence of sleep problems, the factors contributing to them, beliefs about causes, and favoured solutions. | An original questionnaire with 23 questions. It is not clear whether options were presented for selection or free text responses entered and then analyses quantitatively | Sleep problems perceived to be since: an event (38%), a condition (25%), since the participant remembers (23%), a certain medication (15%). The majority slept more than 6 hours, and the majority felt they were not getting enough sleep. 10% reported needing over 10hrs. Most felt sleep disturbances impacted on mood, but many fewer reported effects on social or work functioning. Few reported using non-pharmacological approaches, 13% reported doing nothing. |
| **Zimmermann et al. 2013**  *Patient preferences for outcomes of depression treatment*  (Germany) | 227 outpatients.  Depression, 61.2% receiving long-term or recurrent treatment.  38% male. | To evaluate patient’s perspectives on what are the most troubling symptoms of depression and which are more important to treat. | Survey, participants rank case studies using Choice-based conjoint analysis (case studies have different combinations of symptoms and are rated as being in a better or worse state.) | *Sleep disturbance* was ranked 5^th^ out of 8 symptoms, less important than loss of energy/fatigue, side effects, or loss of interest and enjoyment of activities, but more important than depressed mood, duration of treatment and feelings of guilt. The most important symptom as ranked was loss of energy and fatigue (which would in some but not all cases may be caused by poor sleep.) |
