# Supplementary material for: Perspectives on Sleep, Sleep Problems, and Their Treatment, in People with Serious Mental Illnesses: A Systematic Review
Source: PLoS One. 2016 Sep 22;11(9):e0163486. doi: 10.1371/journal.pone.0163486 (PMC5033349; doi:10.1371/journal.pone.0163486)
Supplement: S3 Table — (DOCX) [file pone.0163486.s006.docx]

**Critical appraisal summaries - qualitative studies**

| **Author/date** | **Sample** | **Data collection** | **Qualitative analysis** | **Ethics and reflexivity** |
| --- | --- | --- | --- | --- |
| **Collier et al. 2003** | MEDIUM | MEDIUM | MEDIUM | POOR |
| Davis & O’Neill 2005 | GOOD | GOOD | GOOD | POOR |
| Engqvist et al. 2011 | MEDIUM | GOOD | GOOD | GOOD |
| Engqvist & Nilsson 2013 | MEDIUM | GOOD | MEDIUM | GOOD |
| **Holmes et al. 1995** | MEDIUM | UNCLEAR | POOR | POOR |
| **MacDonald et al. 2015** | POOR | MEDIUM | MEDIUM | MEDIUM |
| Samalin et al. 2014 | GOOD | MEDIUM | MEDIUM | UNCLEAR |
| **Waite et al. 2015** | GOOD | GOOD | GOOD | GOOD |
| **Waters et al. 2015** | GOOD | POOR | MEDIUM | MEDIUM |

* **Bold text** for author/date = sleep related perspectives were a primary focus, non-bold text for author/date = sleep related perspectives were secondary aim, presented amongst many other findings.

**Critical appraisal summaries - quantitative studies**

| **Author/date** | **Sample** | **Validity and reliability of measures** | **Administration** | **Analysis and interpretation** |
| --- | --- | --- | --- | --- |
| Auslander & Jeste 2002 | MEDIUM | GOOD | GOOD | MEDIUM |
| **Chiu et al. 2015** | MEDIUM | MEDIUM | UNCLEAR | GOOD |
| Harvey et al. 2005 | MEDIUM | MEDIUM | GOOD | GOOD |
| **Li et al. 2011** | GOOD | GOOD | UNCLEAR | MEDIUM |
| Lien et al. 2003 | POOR | POOR | UNCLEAR | POOR |
| Pandina et al. 2010 | MEDIUM | MEDIUM | MEDIUM | GOOD |
| **Peacey et al. 2012** | GOOD | MEDIUM | MEDIUM | MEDIUM |
| Plante et al. 2013 | GOOD | GOOD | UNCLEAR | GOOD |
| Poulin et al.  2010 | GOOD | POOR | GOOD | POOR |
| Mueser et al. 1992 | GOOD | MEDIUM | GOOD | MEDIUM |
| **Niet De et al. 2008** | GOOD | GOOD | GOOD | GOOD |
| **Sobieraj et al. 2013** | MEDIUM | POOR | UNCLEAR | POOR |
| Zimmerman et al. 2013 | MEDIUM | GOOD | GOOD | GOOD |

* **Bold text** for author/date = sleep related perspectives were a primary focus, non-bold text for author/date = sleep related perspectives were secondary aim, presented amongst many other findings.
